# Modulação Autonômica Cardíaca é Fator Chave para Pressão Alta em Adolescentes

**DOI:** 10.36660/abc.20200093

**Published:** 2021-10-06

**Authors:** Sara Raquel Dutra Macêdo, Antonio Carlos Silva-Filho, Adeilson Serra Mendes Vieira, Nivaldo de Jesus Soares, Carlos José Dias, Carlos Alberto Alves Dias, Antonio Woodson Maciel, Luís Gustavo Dias Rabelo, Flavio Oliveira Pires, Rachel Melo Ribeiro, Bruno Rodrigues, Cristiano Teixeira Mostarda

**Affiliations:** 1 Universidade Federal do Maranhão São LuísMA Brasil Universidade Federal do Maranhão, São Luís, MA - Brasil; 2 Faculdade Uninassau São LuísMA Brasil Faculdade Uninassau, São Luís, MA - Brasil; 3 Universidade Estadual de Campinas CampinasSP Brasil Universidade Estadual de Campinas, Campinas, SP - Brasil

**Keywords:** Doenças Cardiovasculares, Pressão Arterial, Hipertensão, Pré-Hipertensão, Obesidade, Adolescente

## Abstract

**Fundamento:**

O interesse pela hipertensão em crianças e adolescentes aumentou desde a atualização do sistema de classificação da pressão arterial para comparar com o sistema de classificação dos adultos, alterando a terminologia de “normal alta” para “pré-hipertensão”.

**Objetivo:**

O objetivo deste estudo foi analisar a associação da modulação autonômica cardíaca com os níveis pressóricos dos adolescentes.

**Métodos:**

203 adolescentes foram agrupados de acordo com a pressão arterial sistólica (PAS) e a pressão arterial diastólica (PAD). Um grupo foi caracterizado como pré-hipertensão, e o outro como normotenso. Foram coletadas características antropométricas, cardiovasculares e de qualidade do sono. Inicialmente, os dados foram submetidos ao teste de normalidade *Kolmogorov-Smirnov* . As variáveis quantitativas contínuas foram analisadas por meio do teste T de *Student* não pareado. Para análise das variáveis categóricas, foi utilizado o teste qui-quadrado. Um modelo de regressão logística foi realizado. O nível de significância estabelecido foi p<0,05. Os dados foram expressos como média ± desvio padrão e intervalo de confiança. O *software R* foi utilizado para análise dos dados. O tamanho do efeito foi calculado com a fórmula de Cohen.

**Resultados:**

O grupo pré-hipertensão apresentou aumento da entropia de Shannon e diminuição da variância total. Além disso, no modelo de regressão logística, os adolescentes deste grupo tiveram 1,03 mais chances de ter a entropia de Shannon afetada quando a PAS foi ajustada ao gênero, maturação sexual, tempo escolar, idade, circunferência da cintura e qualidade do sono.

**Conclusão:**

Nossos dados mostram que a modulação autonômica pode desempenhar um papel importante no desenvolvimento da pressão arterial elevada em adolescentes ao controlar fatores como tempo escolar e qualidade do sono.

## Introdução

No mais recente relatório sobre as causas globais de morte, as doenças cardiovasculares novamente lideraram a mortalidade por todas as causas para doenças não transmissíveis,^1^ sendo a hipertensão uma das principais causas nesta categoria. Devido ao aumento de mortes relacionadas às doenças cardiovasculares, a triagem para o desenvolvimento dessas doenças cresceu e está sendo direcionada à população mais jovem.^2^

O interesse pelo tema da hipertensão em crianças aumentou desde a publicação, em 2004, do Grupo de Trabalho Nacional sobre Educação em Pressão Alta ( *National High Blood Pressure Education Program Working Group),* que atualizou o sistema de classificação da pressão arterial em crianças e adolescentes para que fosse comparável ao sistema de classificação de adultos, mudando a terminologia de “normal alta” para “pré-hipertensão”.^3^

A classificação da pré-hipertensão foi criada para alertar os médicos e justificar a introdução de mudanças de estilo de vida entre aqueles que podem correr o risco de desenvolver hipertensão ainda jovens. Atualmente, sabe-se que crianças com pressão alta têm mais chances de desenvolver hipertensão na vida adulta.^4^ Além disso, estudos recentes sugerem que crianças com pré-hipertensão podem desenvolver a hipertensão até antes de atingirem a vida adulta.^5^

Reconhecendo as lacunas e a necessidade de uma revisão de literatura atualizada e relevante, um estudo de 2020 enfatizou que o diagnóstico da hipertensão em crianças e adolescentes – diferentemente dos adultos – baseia-se em suas curvas de idade e altura.^6^ Assim, é importante ressaltar que, em 2017, a Academia Americana de Pediatria (AAP) e seu Conselho de Desenvolvimento e Segurança do Paciente criaram diretrizes práticas para fornecer informações atuais sobre tópicos relevantes para o diagnóstico, avaliação e tratamento da hipertensão em crianças e adolescentes. Agora, para crianças com idade ≥ 13 anos, a pressão alta é caracterizada de 120/80 até 129/80 mmHg; hipertensão estágio 1, 130/80 até 139/89; hipertensão estágio 2, ≥140 / 90 mmHg.^7^

O sistema nervoso autônomo tem papel importante na rápida regulação da pressão, e tem sido estudado em relação à sua função no desenvolvimento da hipertensão.^8^ Um dos principais fatores correlacionados com a hipertensão é o desequilíbrio autonômico, que também está ligado ao risco cardiovascular.^8 , 9^

Uma das ferramentas mais comuns para avaliar a modulação autonômica é a variabilidade da frequência cardíaca (VFC), especialmente por conta de sua abordagem não-invasiva e grande confiabilidade.^4 - 9^ Entre as avaliações de VFC, as não-lineares medem a complexidade do sinal do eletrocardiograma, que se destaca em relação aos métodos mais comuns de VFC, como o domínio da frequência, que pode ser afetado por sons. A entropia de Shannon é um indicador da complexidade do sinal, e está fortemente relacionada com a modulação autonômica. Porém, os adolescentes também são afetados por outros fatores de risco cardiovasculares, como obesidade^5 - 10^ e baixa qualidade do sono devido à rotina escolar.^11 , 12^ Assim, é importante determinar a contribuição real do desequilíbrio autonômico para o desenvolvimento de altos níveis de pressão arterial. Este estudo teve como objetivo avaliar a associação entre a modulação autonômica cardíaca e a pressão alta em adolescentes.

## Métodos

### Amostra

O tamanho da amostra deste estudo foi estabelecido com base em cálculos estatísticos, utilizando o software G*Power, considerando o teste t bicaudal, com tamanho do efeito (TE) de 0,30, intervalo de confiança de 95%, e probabilidade de erro de 5%. Para essas características, foram encontrados tamanho de amostra de 134 participantes e poder de 0,95.

O presente estudo, então, foi realizado com 203 adolescentes (70 meninos e 133 meninas), com idades entre 11 e 18 anos, agrupados de acordo com a pressão arterial sistólica (PAS) e a pressão arterial diastólica (PAD). Um grupo foi caracterizado como “pré-hipertensão” (44 adolescentes; PAS> 120/80 mmHg), e o outro como “normotenso” (159 adolescentes; PAS<120/80 mmHg). Todos os participantes foram selecionados em uma escola pública do Estado (Centro Anil Rio Anil – CINTRA), em São Luís, Maranhão, Brasil. Todos os métodos utilizados neste estudo foram aprovados pelo comitê institucional de ética e seguiram as diretrizes da Declaração de Helsinki.

### Medidas antropométricas

Inicialmente, as medidas de peso, altura e circunferência da cintura foram feitas de acordo com o Instituto Nacional do Coração, Pulmões e Sangue ( *National Heart, Lung and Blood Institute* ), como descrito previamente.^13^ Escolhemos o método de Slaughter (1988), que utiliza somente as dobras cutâneas tricipital e subescapular para estimar a gordura corporal em adolescentes.^14^

### Maturação sexual

Para determinar a idade biológica da amostra, utilizamos a escala de Tanner autoaplicada, como descrito anteriormente.^15 , 16^

### Qualidade do Sono

A qualidade do sono e a presença de distúrbios do sono foram avaliadas usando o Índice de Qualidade do Sono de Pittsburgh, como originalmente descrito por de Buysse.^17^ A pontuação de cada componente foi incluída para oferecer uma pontuação geral, variando de 0 a 21 pontos. Quanto maior o valor obtido, pior a qualidade do sono (escore global varia de seis a 21).

### Pressão Arterial

A pressão arterial (PA) foi verificada por meio de um monitor automático de pressão arterial (OMRON, HEM-7200, São Paulo, Brasil), validado para medição de pressão arterial em adolescentes.^18^ Os protocolos utilizados para análise da PA se basearam na 7ª Diretriz Brasiliera de Pressão Arterial^19^ e no Quarto Relatório sobre Diagnóstico, Avaliação e Tratamento de Pressão Arterial em Crianças e Adolescentes,^19^ incluindo manguitos apropriados para a idade de acordo com os percentis de altura.^19^

Os adolescentes foram agrupados de acordo com nível de PAS, seguindo as recomendações do *Clinical Practice Guideline for Screening and Management of High Blood Pressure in Children and Adolescents* ^7^ e na Diretriz Brasileira de Pressão Arterial.^19^ Assim, os adolescentes foram divididos em dois grupos, de acordo com valores de PAS e PAD:

Normotenso (n=159): adolescentes saudáveis com PAS 100-119 mm Hg e PAD 60-79 mm Hg.

Pré-hipertensão (n=44): adolescentes saudáveis com PAS 120-139 mm Hg e PAD 80-89 mm Hg.

### Variabilidade da Frequência Cardíaca

A VFC foi analisada utilizando um eletrocardiograma de 12 leads desenvolvido para coletar dados de VFC (Micromed Biotecnologia, Wincardio). Os dados foram obtidos com os participantes na posição supino por 10 minutos. Depois da coleta de dados, os intervalos R-R foram avaliados com o software Kubios (Kuopio, Finlândia), usando a transformada rápida de Fourier, como descrito anteriormente.^20^ Utilizamos tempo (frequência cardíaca (bpm); média RR (ms); SDNN (ms); rMSSD (ms); pNN50 (%); variância total (ms^2^) e domínios não-lineares (SD1 (ms); SD2 (ms); e a entropia de Shannon).

### Análise estatística

Inicialmente, os dados foram submetidos ao teste de Kolmogorov-Smirnov para avaliar a normalidade da sua distribuição. Depois, uma análise descritiva foi realizada e os dados foram expressos em média, desvio padrão e intervalo de confiança para características de base e variáveis contínuas; e como frequência absoluta e porcentagens para variáveis categóricas. O teste T de Student não pareado foi escolhido para analisar as variáveis quantitativas contínuas com distribuição normal. As variáveis categóricas foram analisadas com o teste qui-quadrado. A regressão logística foi realizada para entender qual variável estaria mais associada à pressão arterial na amostra. As estimativas para tal associação foram expressas em razão de chances ( *odds ratio* – OR) e intervalos de confiança de 95% (IC95%). O software R foi usado para a análise de dados. O tamanho de efeito foi calculado de acordo com a fórmula de Cohen.^21^ O nível de significância estabelecido para este estudo foi p <0,05.

## Resultados

O estudo foi realizado com 203 adolescentes agrupados de acordo com seus níveis de PAS e PAD. Um grupo se caracterizou como pré-hipertensão (44 adolescentes; PAS de 120/80 até 129/80 mmHg), e o outro como normotenso (159 adolescentes; PAS<120/80 mmHg).

A Tabela 1 mostra a análise das características antropométricas, cardiovasculares e de qualidade de sono dos adolescentes. Não houve diferenças estatísticas entre os grupos em relação à idade, altura, peso, qualidade do sono, circunferência da cintura, percentual de gordura e escala de Tanner. Porém, houve diferença estatística e um tamanho de efeito significativo para PAS (TE = 0,83) e PAD (TE = 3,05) no grupo pré-hipertensão em comparação ao grupo normotenso ( Tabela 1 ).

**Tabela 1:** – Análise de características antropométricas, cardiovasculares e qualidade do sono em grupos de adolescentes normotensos e com pré-hipertensão

	Normotenso (n= 159)	Pré-hipertensão (n= 44)	∆ (IC)	Valor de p	TE
Idade (anos)	16,00 ± 2,08	16,00 ± 1,71	0,00 (-0,67/0,67)	1,00	0,00
Altura (cm)	161,38 ± 12,19	162,32 ± 9,90	0,94 (-3,00/4,88)	0,63	0,08
Peso (kg)	58,09 ± 10,94	56,74 ± 12,07	-1,35 (-5,10/2,40)	0,47	0,11
Circunferência da cintura (cm)	68,60 ± 7,05	71,00 ± 9,46	2,40 (-0,16/4,96)	0,06	0,28
Gordura corporal (%)	25,12 ± 8,35	25,10 ± 9,70	-0,02 (-2,92/2,88)	0,98	-0,002
Pressão Arterial Sistólica (mmHg)	107,00 ± 7,10	129,50 ± 7,60	20,08 (20,08/24,92)	0,0001*	3,05
Pressão Arterial Diastólica (mmHg)	64,00 ± 7,02	73,00 ± 7,33	9,00 (6,62/11,38)	0,0001*	1,25
Índice de Qualidade do Sono de Pittsburgh	1,86 ± 1,19	2,04 ± 1,76	0,18 (-0,26/0,62)	0,42	0,11

PA: pressão arterial; *p<0,05 vs normotenso; IC: intervalo de confiança; TE: tamanho do efeito.

Em relação às análises das variáveis qualitativas do estudo, observamos que somente o sexo dos adolescentes apresentou diferença estatística significativa, considerando a maior prevalência de meninos com pré-hipertensão, como demonstrado na Tabela 2 .

**Tabela 2:** – Análise de fatores associados à pressão arterial

	Normotenso n=159 (%)	Pré-hipertensão n=44 (%)	χ ^2^
**Sexo**			
Masculino	44 (27,68)	26 (59,10)	[15,0575] p= 0,0001
Feminino	115 (72,32)	18 (40,90)	
**Histórico familiar de hipertensão**			
Pais hipertensos	39 (24,57)	17 (38,65)	[3,4338] p= 0,6387
Pais normotensos	120 (75,43)	27 (61,35)	
**Período escolar**			
Manhã	72 (45,29)	20 (45,46)	[0,0004] p= 0,9838
Tarde	87 (54,71)	24 (54,54)	
**Escala de Tanner**			
1	0 (0,00)	0 (0,00)	[5,1891] p=0,1584
2	17 (8,09)	6 (2,67)	
3	121 (59,32)	29 (14,00)	
4	14 (8,61)	9 (4,15)	

*χ ^2^ teste qui-quadrado; [valor qui-quadrado].*

Dados sobre a modulação autonômica cardíaca nos adolescentes são demonstrados na Tabela 3 . No domínio tempo, foi possível observar que somente a variância total (ms^2^) mostrou diferença estatística e um importante tamanho de efeito. Adolescentes com pré-hipertensão tinham variância total menor (TE = 0,87) em comparação aos normotensos. No domínio não-linear, somente a entropia de Shannon demonstrou diferença estatisticamente significativa e um importante tamanho de efeito (TE = 1,88).

**Tabela 3:** – Dados de modulação autonômica cardíaca em adolescentes com pressão alta e normotensos

	Normotenso (n= 159)	Pré-hipertensão (n= 44)	∆ (IC)	Valor de p	TE
**Medidas de tempo para a variabilidade da frequência cardíaca**					
Frequência cardíaca (bpm)	77,85 ± 12,74	79,91 ± 16,48	2,06 (-2,51/6,37)	0,37	0,14
Média RR (ms)	801,54 ± 110,51	806,99 ± 161,58	5,45 (-35,94/46,84)	0,79	0,03
SDNN (ms)	55,72 ± 27,26	50,96 ± 18,84	-4,76 (-13,39/3,78)	0,27	0,20
RMSSD (ms)	53,10 ± 27,01	48,06 ± 24,03	-5,04 (-13,90/3,82)	0,26	0,19
pNN50 (%)	32,28 ± 20,99	28,47 ± 21,64	-3,81 (-10,90/3,28)	0,29	0,17
Variância total (ms^2^)	3104,71 ± 743,10	2596,92 ± 354,94	-507,79 (-735,85/-279,83)	0,0001*	0,87
**Análise não-linear**					
SD1 (ms)	35,52 ± 15,17	39,65 ± 28,51	4,64 (-26,63/35,91)	0,19	0,18
SD2 (ms)	71,92 ± 100,62	76,56 ± 57,72	5,30 (-25,54/36,14)	0,77	0,05
Entropia de Shannon	1,74 ± 0,27	2,68 ± 0,65	0,94 (0,81/1,06)	0,0001*	1,88

SDNN: Desvio Padrão de todos os intervalor RR normais registrados em um intervalo de tempo; RMSSD: raiz quadrada média de diferenças sucessivas; pNN50 proporção de NN50 dividida pelo número total de NNs; SD1: a dispersão dos pontos perpendiculares à linha de identidade é uma escala de registro instantâneo da variabilidade da frequência cardíaca; SD2: dispersão dos pontos ao longo da linha de identidade; representa a VFC em registros de longo prazo; Valores são apresentados como média ± erro padrão; *p<0,05 vs normotenso; IC: intervalo de confiança; TE: Tamanho do efeito.

Além disso, por meio do modelo de regressão logística, os adolescentes com pré-hipertensão tinham 1,03 mais chances de ter um impacto na entropia de Shannon quando a PAS foi ajustada por sexo, maturação sexual, período escolar, idade, circunferência da cintura e qualidade do sono. Porém, as outras variáveis não demonstraram associação, como demonstrado na Tabela 4 .

**Tabela 4:** – Modelo de regressão logística da associação entre pressão arterial e modulação autonômica

	Entropia de Shannon	Variância total	GOF p
PAS^a^ (OR, IC95%)	1,03 (1,02 – 1,04)	1,01 (0,98 – 1,03)	1,0000
PAD^a^ (OR, IC95%))	0,98 (0,97 – 1,00)	1,02 (0,99 – 1,02)	1,0000

*Pressão arterial sistólica (PAS) e pressão arterial diastólica (PAD) foram ajustadas para sexo, maturação sexual, período escolar, idade, circunferência da cintura, qualidade do sono e histórico familiar de hipertensão; GOF: teste de Hosmer-Lemeshow para avaliar a qualidade do teste; OR: razão de chance; IC95%: intervalo de confiança.*

## Discussão

O objetivo deste estudo foi analisar a associação entre a modulação autonômica cardíaca e os níveis de pressão arterial em adolescentes. Os principais achados deste estudo mostram que os adolescentes no grupo pré-hipertensão tiveram aumento na entropia de Shannon e redução na variância total. Além disso, no modelo de regressão logística, a entropia de Shannon foi a variável que mais esteve associada a mudanças no padrão da pressão arterial, mostrando que adolescentes no grupo pré-hipertensão tinham 1,03 mais chances de ter a entropia de Shannon afetada quando a PAS era ajustada por sexo, maturação sexual, período escolar, idade, circunferência da cintura e qualidade do sono.

Nossos dados corroboram os achados de outros estudos,^22 , 23^ trazendo mais evidências para demonstrar que o aumento da PA entre adolescentes está associado ao desequilíbrio autonômico observado pela variância total menor e pela entropia de Shannon maior. Esses achados são altamente relevantes clinicamente, pois explicam a fisiopatologia da hipertensão, já que mudanças no padrão da VFC promovem um indicador sensível e precoce do distúrbio de saúde. Como descrito por McCraty et al., a VFC alta é sinal de boa adaptação cardiovascular, caracterizando um indivíduo saudável, com mecanismos autonômicos eficientes. Por outro lado, a baixa VFC costuma ser indicativa de adaptação anormal e insuficiente do barorreflexo, o que pode indicar a presença de disfunção autonômica.^24^

Além disso, o estudo de Farah et al.^25^ demonstrou que a VFC pode ser afetada por fatores como sexo, idade, circunferência da cintura e qualidade do sono, o que corrobora outros achados pré-existentes na literatura.^26 , 27^ Porém, de acordo com nosso estudo, o grupo pré-hipertensão não mostrou nenhuma diferença significativa na análise das seguintes variáveis: idade, altura, peso, circunferência da cintura, percentual de gordura e qualidade do sono, o que exclui a possibilidade de esses fatores, isoladamente, influenciarem a VFC. Porém, no modelo de regressão logística, esses fatores associados estiveram relacionados a mais chances de afetar a entropia de Shannon.

O estudo de Amara et al.^28^ publicou mais evidências para apoiar o fato de que o histórico familiar de hipertensão é um importante fator que influencia a VFC, até 30%, e que a hipertensão é duas vezes mais frequente em pessoas que tem um ou dois pais hipertensos. Neste estudo, o histórico familiar de hipertensão provavelmente não mostrou nenhuma relação com as mudanças na VFC devido à ausência de fatores como perfil genético dos adolescentes e a presença de polimorfismos, o que pode influenciar a expressão e a produção de componentes regulatórios que estão presentes no sistema endócrino, como o sistema renina-angiotensina-aldosterona.

Então, embora os mecanismos exatos envolvidos no desenvolvimento da pré-hipertensão em adolescentes não tenham sido plenamente elucidados, estudos recentes, como os de Pal et al. e Wu et al., demonstraram uma associação entre o desequilíbrio autonômico e a menor modulação vagal cardíaca com a pré-hipertensão.^29 , 30^ Outros estudos corroboram esses achados, reportando que a redução na modulação vagal cardíaca e a VFC são as principais causas do maior risco cardiovascular em adolescentes com pré-hipertensão; além disso, obesidade, estresse psicossocial e dislipidemia foram reportados como importantes fatores de risco para pré-hipertensão e hipertensão.^29 - 31^

Atualmente, de acordo com vários estudos,^2 - 8^ o desequilíbrio autonômico tem sido considerado essencial para a etiologia da hipertensão. Em adolescentes, este desequilíbrio autonômico é causado principalmente pela atividade aumentada do sistema nervoso simpático, que também esteve associada ao maior risco de eventos cardiovasculares, obesidade e disfunções do sono.^17 - 26^ Porém, embora a hiperatividade simpática esteja relacionada a outros fatores, como obesidade e problemas no sono, nossos dados não mostraram diferenças na qualidade do sono entre os grupos.

Com relação ao sexo dos adolescentes, alguns estudos observaram que meninos têm maior prevalência de PA alta do que meninas.^32 , 33^ Nesse sentido, os estudos de Sztajzel et al. e Jackson et al., utilizando monitorização ambulatorial de pressão arterial em crianças, mostraram que a idade traz um aumento na pressão arterial tanto para meninos quanto meninas. Porém, depois do início da puberdade, a pressão arterial em meninos é maior do que em meninas da mesma idade. Isso se deve ao fato de jovens mulheres terem tônus vagal cardíaco maior durante o dia e a noite em relação a homens da mesma faixa etária.^34 , 35^

Nosso estudo teve limitações, como a ausência de uma medida direta da qualidade do sono (polissonografia) e um número limitado de participantes, já que é a fase preliminar do estudo. Apesar dessas limitações, conseguimos avaliar a associação da modulação autonômica cardíaca e os diferentes níveis de pressão nos adolescentes.

## Conclusão

Nossos dados mostram que a modulação autonômica pode ter um papel no desenvolvimento da pressão alta, quando controlada por outros fatores, como tempo escolar e qualidade do sono. Este achado tem uma aplicação importante, considerando que o desequilíbrio autonômico pode ser um sinal precoce do desenvolvimento de altos níveis de pressão arterial em adolescentes.
