# Supplementary material for: Modulação Autonômica Cardíaca é Fator Chave para Pressão Alta em Adolescentes
Source: Arq Bras Cardiol. 2021 Oct 6;117(4):648–54. [Article in Portuguese] doi: 10.36660/abc.20200093 (PMC8528363; doi:10.36660/abc.20200093)
Supplement: Translation

# Translation: Cardiac Autonomic Modulation is a Key Factor for High Blood Pressure in Adolescentes

**Authors:** Sara Raquel Dutra Macêdo, Antonio Carlos Silva-Filho, Adeilson Serra Mendes Vieira, Nivaldo de Jesus Soares, Carlos José Dias, Carlos Alberto Alves Dias, Antonio Woodson Maciel, Luís Gustavo Dias Rabelo, Flavio Oliveira Pires, Rachel Melo Ribeiro, Bruno Rodrigues, Cristiano Teixeira Mostarda

**Affiliations:** 1 Brazil Universidade Federal do Maranhão, São Luís, MA - Brazil; 2 Brazil Faculdade Uninassau, São Luís, MA - Brazil; 3 Brazil Universidade Estadual de Campinas, Campinas, SP - Brazil

**Keywords:** Cardiovascular Diseases, Blood Pressure, Hypertension, Pre-Hypertension, Obesity, Adolescent

## Abstract

**Background:**

The interest regarding hypertension among children and adolescents has increased since the blood pressure rating system was updated to be compared with the adult rating system, changing the terminology from “normal high” to “prehypertension”.

**Objective:**

This study aimed to analyze the association between cardiac autonomic modulation and pressure levels of adolescents.

**Methods:**

203 adolescents were grouped according to systolic blood pressure (SBP) and diastolic blood pressure (DBP). One group was characterized as prehypertension, and the other as normotensive. Anthropometric, cardiovascular and sleep quality characteristics were collected. Initially, the data were submitted to the Kolmogorov-Smirnov normality test. Continuous quantitative variables were analyzed using the unpaired Student t-test. For the analysis of categorical variables, a chi-square test was used. A logistic regression model was performed. The level of significance was set at p<0.05. The data were expressed as mean ± standard deviation and confidence interval. The R software was used for data analysis. The effect size was calculated using the Cohen’s formula.

**Results:**

The prehypertension group showed an increase in Shannon entropy and a decrease in total variance. Also, in the logistic regression model, adolescents in this group were 1.03 times more likely to have Shannon entropy’s affected when SBP was adjusted for gender, sexual maturation, school time, age, waist circumference, and sleep quality.

**Conclusion:**

Our data show that autonomic modulation may play an important role in the development of elevated blood pressure in adolescents, when controlling for other factors, such as school time and sleep quality.

## Introduction

In the most recent report on the global cause of deaths, cardiovascular diseases once again led the all-cause mortality for non-communicable diseases,^1^ and hypertension was one of the leading causes in this category. Due to the rise of deaths related to cardiovascular diseases, the screening for the development of these diseases has grown and is being addressed to the younger part of the population.^2^

The interest in childhood hypertension has increased since the publication, in 2004, of the National High Blood Pressure Education Program Working Group, which updated the blood pressure classification system in children and adolescents to match the classification system of adults, changing the terminology from “normal high” to “prehypertension”.^3^

The “prehypertension” classification was created to warn physicians and justify the introduction of lifestyle changes in those who may be at risk for the development of hypertension at a young age. Currently, it is known that children with high blood pressure are more likely to develop hypertension in adulthood.^4^ Also, recent evidence suggests that prehypertensive children may develop hypertension even before they reach adulthood.^5^

Recognizing the ongoing evidence gaps and the need for an updated and relevant literature review, a 2020 study highlighted that the diagnosis of hypertension in children and adolescents – in contrast with adulthood – is based on their age percentile curves and height.^6^ Therefore, it is worth mentioning that in 2017 the American Academy of Pediatrics (AAP) and its Council on Quality Improvement and Patient Safety developed practical guidelines to provide updated information on topics that are relevant for the diagnosis, assessment, and treatment of hypertension in children and adolescents. Now, for children aged ≥ 13 years, high blood pressure is characterized as 120/80 to 129/80 mmHg; stage 1 hypertension is 130/80 to 139/89 mmHg; and stage 2 hypertension is ≥140 / 90 mmHg.^7^

The autonomic nervous system plays an important role in the rapid regulation of blood pressure, and has been long investigated regarding its function in the development of hypertension.^8^ One of the most important factors correlated with hypertension is autonomic imbalance, which is also correlated to cardiovascular risk.^8 , 9^

One of the most used tools for the assessment of autonomic modulation is the Heart Rate Variability (HRV), mainly due to its noninvasive approach and great reliability.^4 - 9^ Among all HRV indexes, the nonlinear ones measure the complexity of the ECG signal, standing out from more common HRV methods, such as the Frequency domain, that can be affected by noise. Shannon entropy is an indicator of the complexity of the signal, being widely correlated with autonomic modulation. However, adolescents are also affected by other cardiovascular risk factors, such as obesity^5 - 10^ and poor sleep quality due to school routine.^11 , 12^ Therefore, it is important to determine the real contribution of autonomic imbalance for the development of high blood pressure levels. This study aimed to evaluate the association of cardiac autonomic modulation with blood pressure levels in adolescents.

## Methods

### Sample

The sample size of this study was established based on statistical calculations using the G*power software, taking into account the two-tailed t-test, with an effect size of 0.30, confidence interval of 95% and probability of error of 5%. For these characteristics, a sample size of 134 participants and power of 0.95 were found.

Thus, the present study was conducted with 203 adolescents (70 boys and 133 girls). aged between 11 and 18 years, grouped according to SBP and DBP. One group was characterized as pre-hypertension (44 adolescents; SBP> 120/80 mmHg), and the other was characterized as normotensive (159 adolescents; SBP <120/80 mmHg). All subjects were selected from a state public school (Centro Anil Rio Anil - CINTRA) in São Luís, Maranhão, Brazil. All methods used in this study were approved by the institutional ethics committee and followed the guidelines from the Declaration of Helsinki.

### Anthropometric measurements

At first, measurements of weight, height, and waist circumference were taken according to the National Heart, Lung and Blood Institute, as previously described.^13^ We chose the Slaughter (1988) method, which uses only the tricipital and subscapular skinfolds for body fat estimation in adolescents.^14^

### Sexual maturation

To determine the biological age of the sample, we used the self-applied Tanner’s Sexual Maturation Scale, as described elsewhere.^15 , 16^

### Sleep Quality

Sleep quality and the presence of sleep disturbances were evaluated using the Pittsburgh Sleep Quality Index, as originally described by de Buysse.^17^ The score of each component was added to provide an overall score, ranging from 0 to 21 points. The higher the value obtained, the worse the sleep quality (global score is between six and 21).

### Blood Pressure

Blood pressure (BP) was verified using an automatic blood pressure monitor (OMRON, HEM-7200, São Paulo, Brazil), validated for blood pressure measurements in adolescents.^18^ The protocols used for the analysis of BP were based on the 7^th^Brazilian Guideline of Arterial Hypertension^19^ and the Fourth Report on the Diagnosis, Evaluation, and Treatment of High Blood Pressure in Children and Adolescents,^19^ including an age-appropriate cuff according to height percentiles.^19^

The adolescents were grouped according to their level of SBP, following the recommendations from the Clinical Practice Guideline for Screening and Management of High Blood Pressure in Children and Adolescents^7^ and the Brazilian Guideline of Arterial Hypertension.^19^ Thus, the adolescents were divided into two groups according to SBP and DBP values:

Normotensive (n=159): healthy adolescents with SBP 100-119 mm Hg and DBP 60-79 mm Hg.

Prehypertension (n=44): healthy adolescents with SBP 120-139 mm Hg and DBP 80-89 mm Hg.

### Heart Rate Variability

The HRV was analyzed using a 12-lead electrocardiogram developed for HRV data collection (Micromed Biotecnologia, Wincardio). The data were collected with the subjects in the supine position during a 10-minute period. After data collection, the RR intervals were analyzed with the Kubios software (Kuopio, Finland), using the Fast Fourier Transform, as described earlier.^20^ We used time (Heart rate (bpm); mean RR (ms); SDNN (ms); RMSSD (ms); pNN50 (%) and total variance (ms^2^) and nonlinear domains (SD1 (ms); SD2 (ms) and the Shannon entropy).

### Statistical analysis

Initially, the data were submitted to the Kolmogorov-Smirnov test to assess the normality of its distribution. Then, a descriptive analysis was conducted and the data were expressed as mean, standard deviation and confidence interval for baseline characteristics and continuous variables, and as absolute frequencies and percentage rates for categorical variables. The unpaired Student t-test was chosen to analyze continuous quantitative variables with normal distribution. Categorical variables were analyzed using the chi-square test. Logistic regression was performed to understand which variable would be best associated with blood pressure in the sample. Point estimates for the associations were expressed as odds ratios (OR) and respective 95% confidence intervals (95%CI). The R software was used for data analysis. The effect size was calculated according to the Cohen’s formula.^21^ The significance level established for this study was p <0.05.

## Results

The study was carried out with 203 adolescents grouped according to their SBP and DBP levels. One group was characterized as prehypertension (44 adolescents; SBP from 120/80 to 129/80 mmHg), and the other as normotensive (159 adolescents; SBP <120/80 mmHg).

Table 1 shows the analysis of the baseline anthropometric, cardiovascular and sleeps quality characteristics of adolescents. No statistical differences were found between groups regarding age, height, weight, sleep quality index, waist circumference, fat percentage, and the Tanner scale. However, there was a statistical difference and a strong effect size for SBP (ES = 0.83) and DBP (ES = 3.05) in the prehypertensive group when compared to the normotensive group ( Table 1 ).

**Table 1:** – Baseline analysis of anthropometric, cardiovascular characteristics and sleep quality in groups of normotensive and prehypertensive adolescents

	Normotensive (n= 159)	Prehypertensive (n= 44)	∆ (CI)	p value	ES
Age (years)	16.00 ± 2.08	16.00 ± 1.71	0.00 (-0.67/0.67)	1.00	0.00
Height (cm)	161.38 ± 12.19	162.32 ± 9.90	0.94 (-3.00/4.88)	0.63	0.08
Weight (kg)	58.09 ± 10.94	56.74 ± 12.07	-1.35 (-5.10/2.40)	0.47	0.11
Waist circumference (cm)	68.60 ± 7.05	71.00 ± 9.46	2.40 (-0.16/4.96)	0.06	0.28
Body fat (%)	25.12 ± 8.35	25.10 ± 9.70	-0.02 (-2.92/2.88)	0.98	-0.002
Systolic Blood Pressure (mmHg)	107.00 ± 7.10	129.50 ± 7.60	20.08 (20.08/24.92)	0.0001*	3.05
Diastolic Blood Pressure (mmHg)	64.00 ± 7.02	73.00 ± 7.33	9.00 (6.62/11.38)	0.0001*	1.25
Pittsburgh Sleep Quality Index	1.86 ± 1.19	2.04 ± 1.76	0.18 (-0.26/0.62)	0.42	0.11

BP: blood pressure; *p<0.05 vs normotensive; CI: confidence interval; ES: effect size.

Regarding the analyses of qualitative variables in the study, we observed that only the sex of the adolescents presented statistical difference, considering the higher prevalence of prehypertensive boys, as shown in Table 2 .

**Table 2:** – Analysis of factors associated with blood pressure

	Normotensive n=159 (%)	Prehypertensive n=44 (%)	χ^2^
**Sex**			
Male	44 (27.68)	26 (59.10)	[15.0575] p= 0.0001
Female	115 (72.32)	18 (40.90)	
**Family history of hypertension**			
Hypertensive parents	39 (24.57)	17 (38.65)	[3.4338] p= 0.6387
Normotensive parents	120 (75.43)	27 (61.35)	
**School time**			
In the morning	72 (45.29)	20 (45.46)	[0.0004] p= 0.9838
In the afternoon	87 (54.71)	24 (54.54)	
**Tanner’s sexual maturation index**			
1	0 (0.00)	0 (0.00)	[5.1891] p=0.1584
2	17 (8.09)	6 (2.67)	
3	121 (59.32)	29 (14.00)	
4	14 (8.61)	9 (4.15)	

*χ^2^Chi square test; [chi square value].*

The data on cardiac autonomic modulation of the adolescents are shown in Table 3 . In the time domain, it was possible to notice that only the total variance (ms^2^) showed statistical difference and a strong effect size. Adolescents with prehypertension had lower total variance (ES = 0.87) when compared to normotensive adolescents. In the non-linear domain, only Shannon entropy showed a statistical difference and a strong effect size (ES=1.88).

**Table 3:** – Cardiac autonomic modulation data of adolescents with high blood pressure and normotensive

	Normotensive (n= 159)	Prehypertensive (n= 44)	∆ (CI)	p value	ES
**Time domain measures of heart rate variability**					
Heart rate (bpm)	77.85 ± 12.74	79.91 ± 16.48	2.06 (-2.51/6.37)	0.37	0.14
Mean RR (ms)	801.54 ± 110.51	806.99 ± 161.58	5.45 (-35.94/46.84)	0.79	0.03
SDNN (ms)	55.72 ± 27.26	50.96 ± 18.84	-4.76 (-13.39/3.78)	0.27	0.20
RMSSD (ms)	53.10 ± 27.01	48.06 ± 24.03	-5.04 (-13.90/3.82)	0.26	0.19
pNN50 (%)	32.28 ± 20.99	28.47 ± 21.64	-3.81 (-10.90/3.28)	0.29	0.17
Total variance (ms^2^)	3104.71 ± 743.10	2596.92 ± 354.94	-507.79 (-735.85/-279.83)	0.0001*	0.87
**Nonlinear analysis**					
SD1 (ms)	35.52 ± 15.17	39.65 ± 28.51	4.64 (-26.63/35.91)	0.19	0.18
SD2 (ms)	71.92 ± 100.62	76.56 ± 57.72	5.30 (-25.54/36.14)	0.77	0.05
Shannon entropy	1.74 ± 0.27	2.68 ± 0.65	0.94 (0.81/1.06)	0.0001*	1.88

SDNN: Standard deviation of all normal RR intervals recorded in a time interval; RMSSD: root mean square of successive differences; pNN50 the proportion of NN50 divided by the total number of NNs; SD1: dispersion of the points perpendicular to the line of identity is an index of instantaneous recording of the variability of beat-to-beat; SD2: dispersion of points along the identity line; represents HRV in long-term records; Values are presented as mean± standard error; *p<0.05 vs normotensive; CI: confidence interval; ES: Effect size.

Also, through the logistic regression model, prehypertensive adolescents presented 1.03 more chances of having an impact on Shannon entropy when SBP was adjusted for gender, sexual maturation, school time, age, waist circumference, and sleep quality. However, the other variables showed no association, as shown in Table 4 .

**Table 4:** – Logistic regression model of the association between blood pressure and autonomic modulation

	Shannon entropy	Total variance	GOF p
PAS^a^(OR, 95% CI)	1.03 (1.02 – 1.04)	1.01 (0.98 – 1.03)	1.0000
PAD^a^(OR, 95% CI)	0.98 (0.97 – 1.00)	1.02 (0.99 – 1.02)	1.0000

*Systolic blood pressure (SBP) and diastolic blood pressure (DBP) were adjusted (a) for sex, sexual maturation, school time, age, waist circumference, sleep quality and family history of hypertension; GOF: Hosmer and Lemeshow’s goodness of fit test; OR: odds ratio; 95% CI: confidence interval.*

## Discussion

This study aimed to analyze the association between cardiac autonomic modulation and blood pressure levels in adolescents. The main findings of the present study show that adolescents in the prehypertension group showed increased Shannon entropy and decreased total variance. Also, in the logistic regression model, Shannon entropy was the variable that was mostly associated with changes in blood pressure pattern, showing that adolescents in the prehypertension group had 1.03 more chances of having Shannon entropy affected when the SBP was adjusted by gender, sexual maturation, school time, age, waist circumference and sleep quality.

Our data corroborate the findings of other studies,^22 , 23^ providing more evidence to show that an increase in BP levels among adolescents is associated with the autonomic imbalance observed by the lower total variance and higher Shannon entropy. These findings are highly clinically relevant for understanding the pathophysiology of hypertension, since changes in HRV patterns provide a sensitive and early indicator of health impairment. As described by McCraty et al., high HRV is a sign of good cardiovascular adaptation, characterizing a healthy individual with efficient autonomic mechanisms. On the other hand, low HRV is generally an indicator of abnormal and insufficient adaptation of the baroreflex, which may indicate the presence of autonomic dysfunction.^24^

In addition, the study by Farah et al.^25^ showed that HRV can be affected by factors including sex, age, waist circumference and sleep quality, which corroborates other pre-existing findings in the literature.^26 , 27^ However, according to our study, the prehypertension group did not show any significant differences in the analysis of the following variables: age, height, weight, waist circumference, fat percentage and sleep quality, which excluded the possibility of these isolated factors influencing HRV. However, in the logistic regression model, these associated factors were related to increased chances of affecting Shannon entropy.

In the study by Amara et al.,^28^ they published more evidence supporting that family history of hypertension is an important factor influencing HRV, up to 30%, and that hypertension is twice as common in individuals who have one or two hypertensive parents. In the present study, the family history of hypertension probably did not show any relationship with changes in HRV due to the absence of factors such as the genetic profile of adolescents and the presence of polymorphisms, which can influence the expression and production of regulatory components that are present in the endocrine system, such as the renin-angiotensin-aldosterone system.

Thus, although the precise mechanisms involved in the development of prehypertension in adolescents have not yet been clearly explained, recent studies, such as the ones by Pal et al. and Wu et al., have demonstrated an association between autonomic imbalance and decreased vagal modulation with prehypertension.^29 , 30^ Other studies corroborate these findings, reporting that the decrease in vagal modulation and HRV are the main causes of increased cardiovascular risk in adolescents with prehypertension; besides, obesity, psychosocial stress and dyslipidemia have been reported as important risk factors for prehypertension and hypertension.^29 - 31^

Currently, according to several studies,^2 - 8^ autonomic imbalance has been widely accepted as being fundamental to the etiology of hypertension. In adolescents, this autonomic imbalance is mainly caused by increased activity of the sympathetic nervous system, which has also been associated with an increased risk of cardiovascular events, obesity and sleep disorders.^17 - 26^ However, although sympathetic hyperactivity is linked to other factors, such as obesity and sleep disorders, our data did not show differences in sleep quality between groups.

Regarding the gender of adolescents, some studies have observed that boys have higher prevalence of high BP than girls.^32 , 33^ In this sense, the studies by Sztajzel et al. and Jackson et al., using ambulatory BP monitoring in children, revealed that with age there is an increase in blood pressure among boys and girls. However, after the onset of puberty, the blood pressure in boys is higher than that of girls at the same age. This is because young women have higher day and night vagal tone than men of similar age.^34 , 35^

Our study faced limitations such as the absence of a direct measure of sleep quality (polysomnography) and a limited number of subjects, as it is a preliminary phase of the study. Despite these limitations, we were able to assess the association of cardiac autonomic modulation with the different pressure levels of adolescents.

## Conclusion

In conclusion, our data show that autonomic modulation may play a role in the development of elevated blood pressure, when controlling for other factors, such as school time and sleep quality. This finding has an important application considering that autonomic imbalance can be an early sign for the development of high levels of blood pressure in adolescents.
